# Learning Models for Traumatic Brain Injury Mortality Prediction on Pediatric Electronic Health Records

**DOI:** 10.3389/fneur.2022.859068

**Published:** 2022-06-10

**Authors:** João Fonseca, Xiuyun Liu, Hélder P. Oliveira, Tania Pereira

**Affiliations:** ^1^Institute for Systems and Computer Engineering, Technology and Science, Porto, Portugal; ^2^Department of Anesthesiology and Critical Care Medicine, Johns Hopkins University, Baltimore, MD, United States; ^3^Faculty of Science, University of Porto, Porto, Portugal

**Keywords:** machine learning, feature selection, feature importance, Traumatic Brain Injury, mortality prediction, clinical significance, intensive care unit

## Abstract

**Background:**

Traumatic Brain Injury (TBI) is one of the leading causes of injury related mortality in the world, with severe cases reaching mortality rates of 30-40%. It is highly heterogeneous both in causes and consequences, complicating medical interpretation and prognosis. Gathering clinical, demographic, and laboratory data to perform a prognosis requires time and skill in several clinical specialties. Machine learning (ML) methods can take advantage of the data and guide physicians toward a better prognosis and, consequently, better healthcare. The objective of this study was to develop and test a wide range of machine learning models and evaluate their capability of predicting mortality of TBI, at hospital discharge, while assessing the similarity between the predictive value of the data and clinical significance.

**Methods:**

The used dataset is the Hackathon Pediatric Traumatic Brain Injury (HPTBI) dataset, composed of electronic health records containing clinical annotations and demographic data of 300 patients. Four different classification models were tested, either with or without feature selection. For each combination of the classification model and feature selection method, the area under the receiver operator curve (ROC-AUC), balanced accuracy, precision, and recall were calculated.

**Results:**

Methods based on decision trees perform better when using all features (Random Forest, AUC = 0.86 and XGBoost, AUC = 0.91) but other models require prior feature selection to obtain the best results (k-Nearest Neighbors, AUC = 0.90 and Artificial Neural Networks, AUC = 0.84). Additionally, Random Forest and XGBoost allow assessing the feature's importance, which could give insights for future strategies on the clinical routine.

**Conclusion:**

Predictive capability depends greatly on the combination of model and feature selection methods used but, overall, ML models showed a very good performance in mortality prediction for TBI. The feature importance results indicate that predictive value is not directly related to clinical significance.

## 1. Introduction

Traumatic Brain Injury (TBI) is an extremely incident condition worldwide (1), accounting for a major reason for morbidity, mortality, disability, and reduced quality of life (2–4). The most recent data from the Center for Disease Control (CDC) reports more than 610 TBI-related hospitalizations and 166 TBI-related deaths per day in the United States (US) (5). In Europe, there are 4,109 hospitalizations and 156 deaths per day related to TBI, with reports of population-normalized data stating a three-fold higher incidence of TBI in Europe than in the US (6). The severity of this condition leads to high mortality rates ranging from 3.3 to 28.1 per 1,00,000 in European countries (7) and, in the US, as high as 36% of hospitalized patients die from TBI-related complications (8). Regarding the pediatric sector, TBI is becoming the major cause of death and disability in children (9). Despite the improvement in healthcare over the recent years, TBI incidence may be increasing worldwide, mainly due to the higher use of motor vehicles and consequent increase in traffic-related accidents (7). Comparing the high-income countries with low- or mid- income countries, the main reasons for TBI are quite different, with falls being the leading cause in the US, whereas, traffic accidents being the number one cause of TBI in China (10, 11).

Previous studies revealed that early treatment is beneficial for TBI recovery. It is of great importance to conduct early interventions and treatment before secondary injury and brain deterioration happen. However, a precise prognosis of TBI outcome is difficult due to the high heterogeneity of the disease, i.e., a wide range of lesions (including a skull fracture, hemorrhage, and laceration) and affected areas of the brain (epidural, subarachnoid, and intraparenchymal) (12, 13). To deal with this variability, many factors are evaluated on the patient: medical signals such as intracranial pressure and blood pressure, annotations such as the Glasgow Coma Scale (GCS) and pupil reactivity but also imaging like computerized tomography (CT) annotations (midline shift, type of hemorrhage, edema). Aggregating all sorts of data to obtain a prognosis is quite cumbersome for medical staff, often requiring many areas of expertise (neurology, imaging, and laboratory). Traditionally, doctors use clinical scores such as GCS to predict patient outcomes. However, the accuracy of GCS depends on the experience of the nurse who is conducting the clinical queries, and it varies between different nurses.

Considering that TBI is often an emergency case, increasing efficiency in data analysis is important. Therefore, many previous studies were dedicated to developing outcome predictors based on machine learning (ML) algorithms (14), which have the capability of fast data analysis and can provide medical staff with a prognosis helping tool. Researchers proved that combining GCS with other variables, including initial intracranial pressure, cerebral auto-regulation index, yields better prediction accuracy of outcome after TBI. In recent years, different machine learning approaches have been introduced to ICU to predict patient outcomes, however, there is no gold standard about which model works best, and which parameters should be extracted as the input.

CRASH (15) and IMPACT (16) are examples of effective ML models for TBI outcome prediction which are publicly available on the corresponding websites, proving the utility of ML in this area. These models focus on 6-month outcome prediction using clinical annotations, imaging, and demographic data. Despite their adequate performance using logistic regression and their meticulous analysis of the predictive value of features, external validation studies have come to the conclusion that these predictive models required maintenance to improve their generalization capacity (17, 18). In addition, these models disregard pediatric patients due to the differences between pediatric and adult head injuries. IMPACT only analyzes patients above 14 years old and CRASH only analyzes patients above 40 years old. Recent literature has proposed that more complex models do not improve the quality of mortality prediction, but the used features greatly influence performance (19). However, non-consensual information has been deposed in other works with ML algorithms such as Artificial Neural Networks showing great performance in mortality prediction, using similar clinical and demographic data (20, 21). In these studies, we often notice a lack of disclosure of hyperparameters and unclarity of the used methods, impeding the reproducibility of results.

Therefore, the defined goal is predicting mortality at discharge of pediatric patients with TBI, using demographic and clinical data as well as CT findings gathered during the patient stay at the hospital. To do so, a variety of models and feature selection processes are explored, to understand the dependence of prediction quality on the used features and model type and which combination of model and feature selection methods works best in this specific cohort of patients. Besides evaluating feature value for the prediction, the coherence with their clinical significance is also discussed in detail. A pediatric patient dataset was selected as the focus of this exploratory study because despite being less studied, it is utterly important to develop medical decision support tools for this group of patients as well. Pediatric TBI has been thoroughly studied but mostly from a clinical and epidemiological perspective (22–24). From a clinical perspective, this age group presents more challenges in prediction tasks since the brain is still under development and the effects of the injury to an area in constant alteration are harder to predict (22). Therefore, this study adds the point of view of computational and predictive analysis of this population (aged between 0 and 14 years) as there is a great need to develop solutions that help physicians in clinical decisions in this specific group of patients. Since the study focuses on a new cohort of patients, a more exploratory approach was chosen, experimenting different combinations of models and feature selection methods.

## 2. Materials and Methods

**Figure 1:**
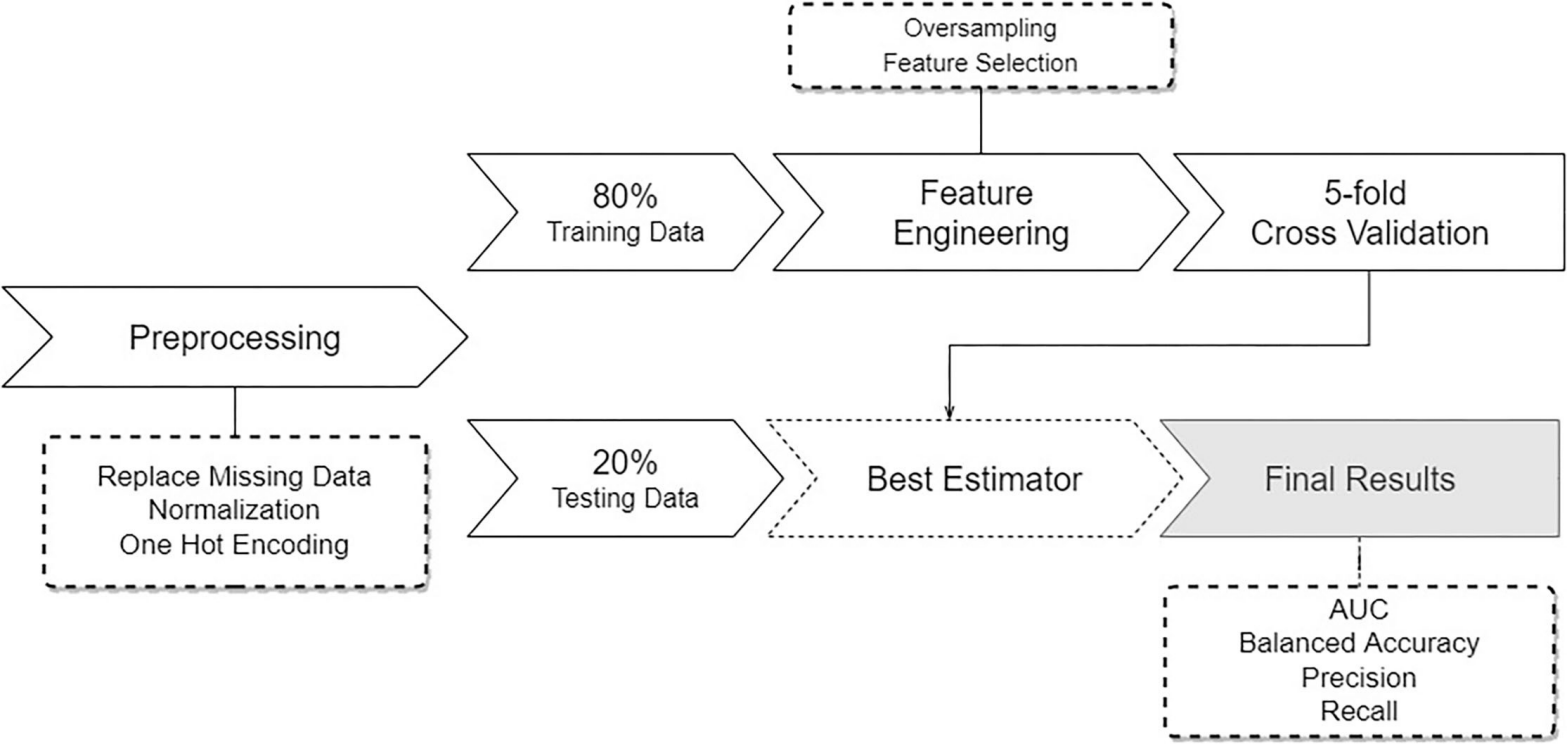
Overview of the pipeline describing the developed approach. Starting with pre-processing of the data, followed by a data split, feature engineering and sampling, and subsequent training and testing several learning models.

The methods used in this study are summarized in Figure 1. The pipeline starts by performing data pre-processing, followed by model training and testing.

### 2.1. Dataset

**Figure 2:**
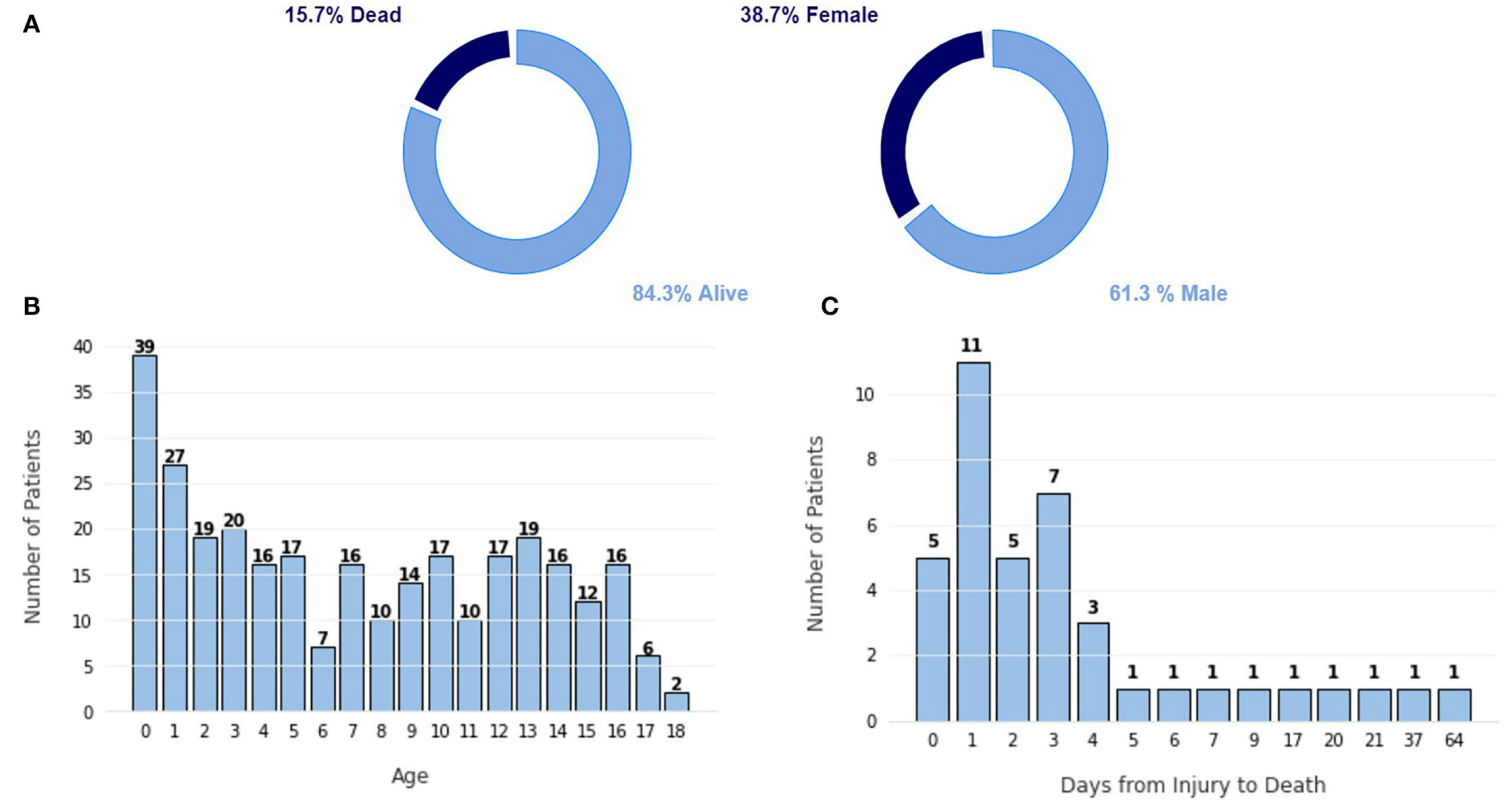
Characterization of the dataset. **(A)** Proportion of class labels, **(B)** Frequency plot of the number of patients per age. **(C)** Frequency plot of the number of patients vs. the number of days since the injury to the death.

The dataset used in this study is the Hackathon Pediatric Traumatic Brain Injury (HPTBI) dataset (25). It comprises 300 hospitalized pediatric patients, of which 84% are alive, as seen in Figure 2A. Sixty-four percent of the patients are male, and the average age is 7.2±5.5 years, with a median of 6.9 years of age.

The highest portion of this cohort is infants under the age of 1 year old, as shown in Figure 2B, likely due to the frail build of infants of that age, which promotes more traumatic injuries. Other age groups show a fairly similar level, promoting a more balanced dataset. In Figure 2C, we can see that most patients die in the first few days after injury because more severe injuries tend to be hard to recover from. Only five patients died after more than 10 days after the injury.

A total of 96 features of different types, summarized in Table 1, were used for the training of the models. Within these features, there are binary features, mostly describing if a certain diagnostic or observation was made (e.g., presence of midline shift in a CT scan), numerical features, accounting for the days between admission and a certain event (e.g., days from admission to tracheostomy), and multi-categorical types, which classify injury mechanism or scales such as the Glasgow Coma Scale (GCS).

**Table 1:** Summary of the different types of data and parameters in the Hackathon Pediatric Traumatic Brain Injury (HPTBI) dataset.

**CT^**a**^ findings**	**Clinical data**	**Demographics**
CT positive for cerebral edema or brain swelling?	Catheter type, quantity, and length of stay	Age
CT positive for compression or effacement of the basilar cisterns?	ICP^b^ type, quantity, and length of stay	Where did the patient go when they left the ED?
CT positive for epidural hematoma?	Did the patient have a cardiac arrest?	Sex
CT positive for intraparenchymal hemorrhage?	Did the patient receive a decompressive craniectomy?	Days from injury to admission
CT positive for intraventricular hemorrhage?	Did the patient receive enteral nutrition?	
CT positive for midline shift?	Did the patient have an epidural hematoma evacuated?	
CT positive for skull fracture?	Cardiac arrest	
CT positive for subarachnoid hemorrhage?	GCS^c^ICU^d^ (eye, motor, verbal, and total)	
CT positive for subdural hematoma?	GCS ED^e^ (eye, motor, verbal, and total)	
	Pharmaceuticals ordered (barbiturate, mannitol, inotrope or vasopressor, hypertonic saline)	

a *Computed Tomography*.

b *Intracranial pressure*.

c *Glasgow Coma Scale*.

d *Intensive Care Unit*.

e *Emergency Department*.

### 2.2. Pre-Processing

First, features related to the Functional Status Scale (FSS) were removed from the data since FSS could not be assessed for patients who perished. Therefore, they are not useful as there is a high correlation between mortality and missing data on these features.

One Hot Encoding (OHE) was performed to transform categorical features into *n* binary variables, where *n* was the number of categories in that variable. Data were normalized by scaling each feature to have a unit norm. After the feature selection methods (Refer to Section 2.3), due to the high imbalance of the dataset and its smaller size, the data were oversampled using Synthetic Minority Oversampling Technique (SMOTE) (26). With SMOTE, synthetic samples of the minority class (mortality) were created to achieve a 50/50 balance of the data.

### 2.3. Feature Selection Methods

Feature selection allows us to decrease data dimensionality, hence reducing computation cost, by removing redundant features or features that contribute little to the predictive capability of the model.

Three methods were tested, starting with a simple feature selection method, using the Koehrsen's Feature Selector (KFS) tool by Koehrsen (27). The first step in Koehrsen's feature selector is removing highly correlated features, i.e., with a Pearson Correlation coefficient above 0.90. From the pair of highly correlated features, one is selected randomly. For the second step, feature importance is computed for all features by a gradient boosting machine (GBM), for 10 iterations. With that information, zero importance and low-importance features, that do not contribute to cumulative importance of 0.95, are removed from the training set. Applying Gradient Boosting Models for feature selection has proven to be of value in previous literature (28–30).

Then, Principal Component Analysis (PCA) and Independent Component Analysis (ICA) were tested to verify if these highly used methods in ML outperform the simpler first approach. PCA and ICA are two extremely different approaches to dimensionality reduction since they provide different feature spaces obtained from the data (31, 32). In order to best understand the impact of the number of PCA components in model performance, it was varied from 3 to 15, corresponding to 0.900 and 0.995 of cumulative variance explained respectively. The same number of components were used in PCA and ICA, in order to maintain a fair comparison.

### 2.4. Classification Models

In this study, a wide range of modest models was presented to establish baseline results and provide a foundation on which to improve. Four machine learning-based methods were implemented in this study, covering different strategies of learning and allowing a heterogeneous analysis of the data since there is no previous knowledge about TBI prediction in the pediatric cohort. For this reason, were implemented methods based on decision trees, neural networks, and clustering. Artificial Neural Networks are tested in this study as it is a highly used method for a variety of tasks, including TBI mortality prediction (20). KNN was also tested to verify how a clustering method performs in this task.

Since one of the objectives of this study is to quantify and qualify the predictive value of features, models such as Random Forest (RF) and eXtreme Gradient Boosting (XGBoost) are useful (20, 33), as they have the inherent capability of computing feature importance, making them good model choices to test in healthcare related tasks. The results of the computation of the feature importance of RF and XGBoost are presented in Section 3.2, along with the feature importance computed by Koehrsen's Feature Selector.

KNN and ANN present limitations when speaking of feature importance, which is one of the reasons that they are inherently harder to interpret. For KNN, the distance between clusters could be used to indirectly obtain feature importance, and for ANN, the weights of the nodes could also be used for similar purposes. However, the methods to achieve explanations for these models are another area of expertise (explainable artificial intelligence, xAI) that falls out of the scope of such a preliminary study. Another approach to make these models more interpretable is to pair them with an external feature selection method, such as Koehrsen's Feature Selector as it is done in this study.

### 2.5. Training

Before training, 20% of the data is held out for the final testing. The remaining 80% is used for training. This proportion was chosen based on previous study (21). To train the models, a five-fold cross-validation (CV) method is applied in order to reduce splitting bias as much as possible. The metric used for refitting the models during CV was the area under the receiver operator characteristic curve (ROC-AUC). Training scores are obtained by averaging the 5 validation set results. Each of the 5 validation results will generate an estimator and we obtain the best estimator out of the 5, for each model. Then, this best estimator will be fitted to the test set and evaluated, before giving us the final test results. The metrics used for evaluation are Balanced Accuracy, Precision, Recall, and AUC. The entire pipeline is run for 50 trials and the results are averaged. In this way, the obtained results are more robust as they are not biased to a specific split (29). To tune the hyperparameters of the models, a grid search was performed, where each parameter was varied within a range. This information is summarized for each model in Table 2.

**Table 2:** Table summarizing the tuned hyperparameters for each model and the corresponding best and most frequent values.

	**Parameter**	**Values**	**Best value**				**Most Frequent value**			
			**KFS^**f**^**	**PCA^**g**^**	**ICA^**h**^**	**No FS^**i**^**	**FS**	**PCA**	**ICA**	**No FS**
KNN^j^	Number of neighbors	1 : 1 : 10	6	9	9	6	7	9	9	8
	Weights	Uniform, distance	Distance	Distance	Distance	Distance	Distance	Distance	Distance	Distance
	Distance metric	Manhattan, Euclidean	Manhattan	Manhattan	Euclidean	Manhattan	Manhattan	Manhattan	Euclidean	Manhattan
RF^k^	Number of estimators	20 : 1 : 50	25	37	37	25	25	37	42	37
	Max depth of the tree	10, 30, 50, 85, 100, None	30	30	85	30	30	30	None	10
	Max features to split	Square root, log2	Square root	Square root	Square root	Square root	log2	Square root	Square root	Square root
	Minimum samples per Leaf	1,2,5,8,10	1	1	1	1	1	1	1	1
	Minimum samples to split	1,2,5,8,10	2	2	2	2	2	2	2	2
ANN^l^	Solver	LBFGS, Stochastic Gradient Descent, ADAM	lbfgs	lbfgs	lbfgs	lbfgs	lbfgs	lbfgs	lbfgs	lbfgs
	Activation function	Identity, logistic, tanH, ReLU	tanH	tanH	tanH	tanH	tanH	tanH	tanH	tanH
	Alpha	0.0001,0.001, 0.01, 0.05, 0.1	0.01	0.001	0.01	0.05	0.01	0.001	0.001	0.01
	Learning rate	Constant, adaptive	adaptive	Constant	Adaptive	Constant	Adaptive	Constant	Adaptive	Adaptive
XGBoost^m^	Number of estimators	50, 100, 1000	100	1,000	100	1,000	1,000	1,000	1,000	1,000
	Max depth	1, 3, 7, 10	7	7	10	7	7	7	7	7
	Subsample	0.3 : 1.0	1	1	1	1	1	1	1	1
	Alpha	0.0001,0.001, 0.01, 0.05, 0.1	0.001	0.001	0.0001	0.001	0.0001	0.0001	0.0001	0.001
	Colsample by tree	0.3 : 1.0	0.5	0.3	0.5	0.3	0.5	0.5	0.5	0.3
	Learning rate	0.001, 0.01, 0.05, 0.1, 1	0.1	0.1	0.1	0.1	0.1	0.1	0.1	0.05

*The best value is the value of the hyperparameters on the best estimator among all 50 trials. The most frequent value is the value that shows up the most among the 50 estimators that are obtained throughout the 50 trials*.

f *Koehrsen's Feature Selector*.

g *Principal Component Analysis*.

h *Independent Component Analysis*.

i *Feature Selection*.

j *k-Nearest Neighbors*.

k *Random Forest*.

l *Artificial Neural Networks*.

m *eXtreme Gradient Boosting*.

## 3. Results and Discussion

### 3.1. Best Hyperparameters

The choice of hyperparameters has always been a major focus of study in machine learning. It is extremely task and data specific which impedes data scientists to define a generalized feature selection method. In this study, a vast range of hyperparameters was tested, as seen in Table 2. Since the pipeline included 50 trials, with each one giving us the best estimator per model, the full pipeline provides a list of 50 estimators per model. The frequency of each hyper-parameter value in this list of the best estimators was studied and the values that did not come up very often were removed. So in Table 2, we present the best estimators among the 50 trials, i.e., the estimator that obtained the highest AUC and the most frequent values, which is the value that shows up more times in the best estimator of each of the 50 trials.

Usually, the best value is also the most frequent one, as seen in Table 2. However, there are some disagreements such as the number of neighbors for KNN, the number of estimators and max depth for RF, and the number of estimators and column samples by the tree for the XGBoost. This is because a small variation in this type of hyperparameters does not influence performance a lot, e.g., 42 and 37 estimators in RF will get similar performance. Therefore, defining the best value for these hyperparameters is difficult, but it gives us an idea of the range of values that are more suited to the task at hand.

On the other hand, other parameters, usually discrete hyperparameters, like solver type and activation functions, are easier to define. For example, the most recommended solver used by ANN is LBFGS as it performs well in small datasets like ours. tanH is also clearly the best activation function for this dataset.

Nevertheless, defining the best hyperparameters can be challenging but what is proposed here is to start with a wide range of values and test the models for a large number of trials, saving the best estimator in each trial. Then, we are able to pick the most often selected hyperparameters and remove the values that are not chosen often. After some iterations of this process, we will get a small range of values that gets similar performances but can run in much less time.

### 3.2. Classification Results

**Figure 3:**
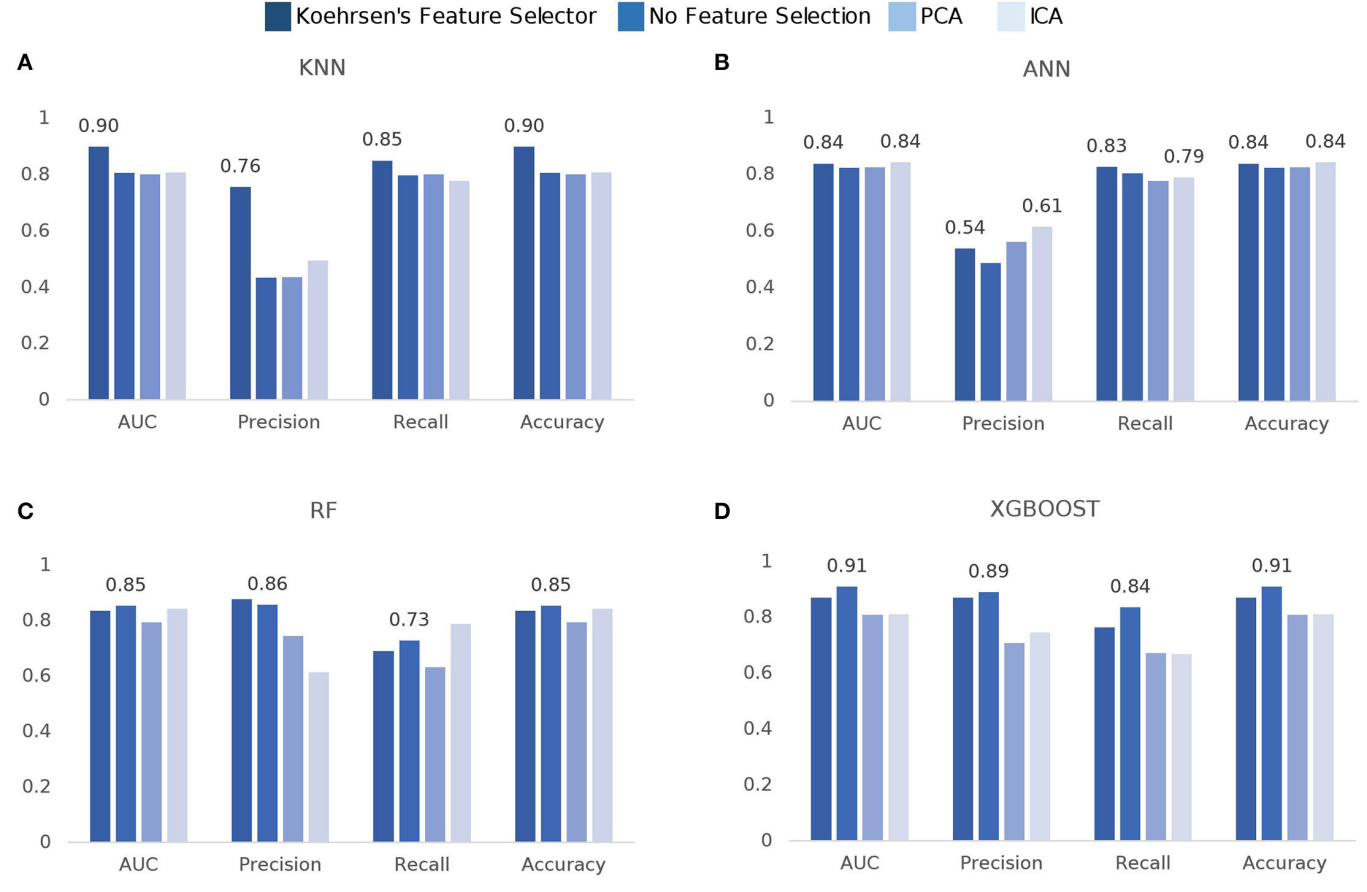
Comparison of model scores per feature selection method: **(A)** k-Nearest Neighbors (KNN), **(B)** Artificial Neural Networks (ANN), **(C)** Random Forest (RF), **(D)** eXtreme Gradient Boosting (XGBoost).

The model comparison is summarized in Figure 3. In terms of ROC-AUC values, XGBoost and KNN are the best performers with 0.91±0.06 and 0.90±0.05 AUC, respectively. XGBoost performs better when no feature selection is used prior to training while KNN performs better when using Koehrsen's feature selector. Since XGBoost is based on decision trees, it was expected that it performed better with no previous feature selection method. The same applies to RF, as its best performance of 0.85±0.08 AUC is achieved with no prior feature selection. For KNN, feature selection has a high impact on the model performance, since it only obtains an AUC of around 0.80 with ICA, PCA, and no feature selection. ANN is the worst performer among the four models, for all metrics. However, it still obtains an AUC value of 0.84±0.08 using ICA with 15 components and Koehrsen's feature selector. Low precision scores indicate a high false-positive rate, i.e., a high mortality prediction, which is naturally incorrect as the class balance of the dataset leans toward non-mortality. The similar performance among all four feature selection methods could be an indication that the features are not important to the model's predictions or that the feature selection methods are not correct. However, since Koehrsen's Feature Selector was able to boost KNN's performance by a considerable margin, the cause of the similar results for all feature selection methods is likely to be the model itself and not the features or feature selection methods. Eventually, a deeper ANN would be able to extract more complex features from the original feature space, which would consequently lead to different results between feature selection methods. With only two hidden layers, the feature spaces extracted will probably be similar between methods, leading to similar results. However, due to the size of the dataset, it would be unwise to use deep networks.

The oversampling of the dataset provided far superior results than using the original unbalanced data. All model score results reported include this processing step prior to the training.

**Figure 4:**
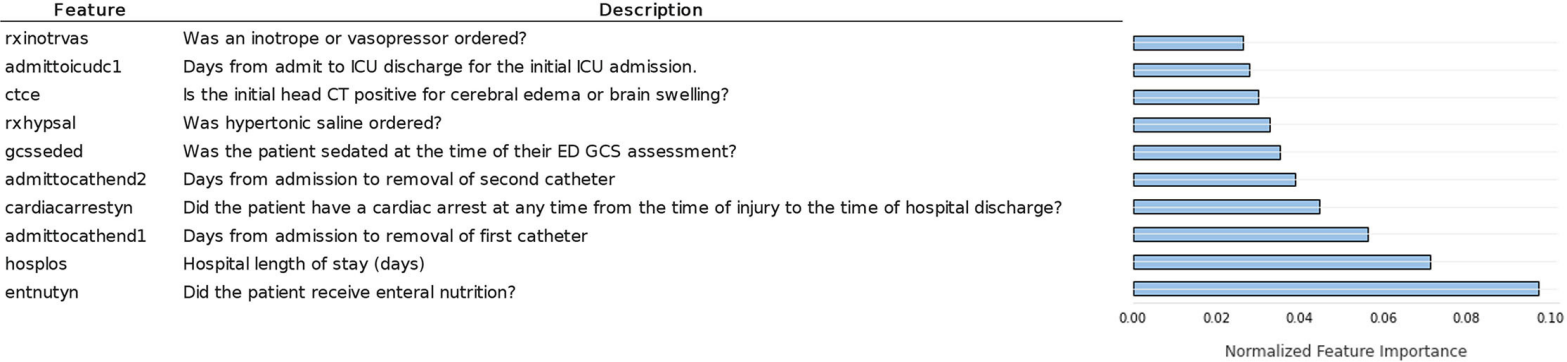
Ten most important features according to normalized feature importance computed by Koerhsen's feature selector tool, which uses a Gradient Boost Model.

**Figure 5:**
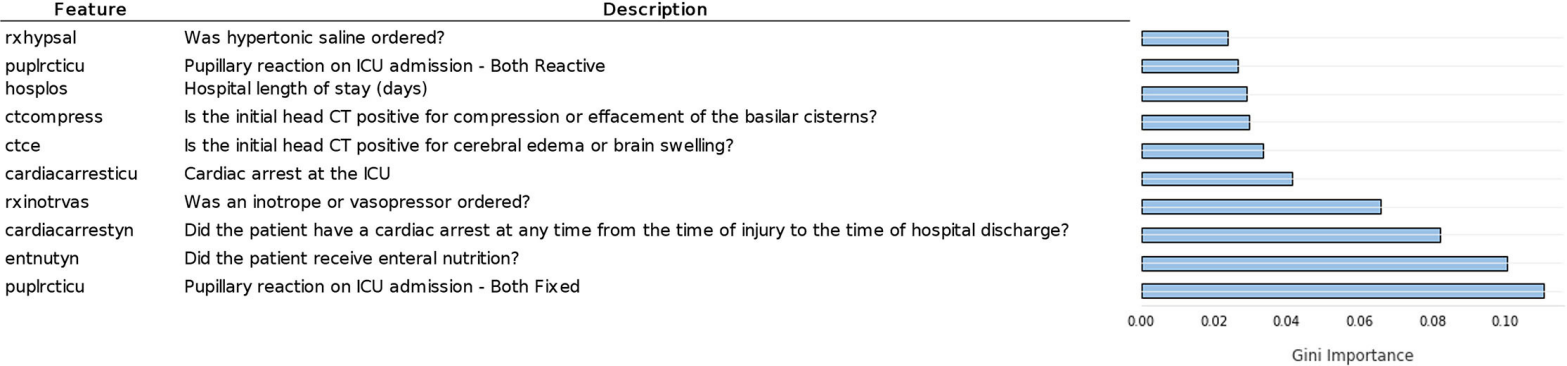
Ten most important features according to Gini feature importance computed by random forest.

Another important topic addressed is the predictive value of the features in this dataset. Feature importance computed by Koehrsen's feature selector is presented in Figure 4. The feature importance has been normalized from 0 to 1 to facilitate interpretation. Feature importance computed by the Random Forest algorithm is seen in Figure 5.

Comparing Figures 4, 5, we notice similarities in the top 10 features of both methods, such as the hospital length of stay, enteral nutrition, pupillary reaction on ICU admission, the existence of a cardiac arrest, brain swelling, or cerebral edema in the CT scan, ordering of inotrope or vasopressors [used in patients in shock to increase cardiac contractility and organ perfusion (34)]. Intuitively, we would grant importance to these features as they are of enormous medical importance, so it was expected our models pick them among the top 10 features. Other features of high predictive value are less coherent with their clinical value such as the number of days from admission to the removal of the first catheter, order of hypertonic saline, and mannitol (commonly used in low severity cases for the nutrition of the patient).

**Figure 6:**
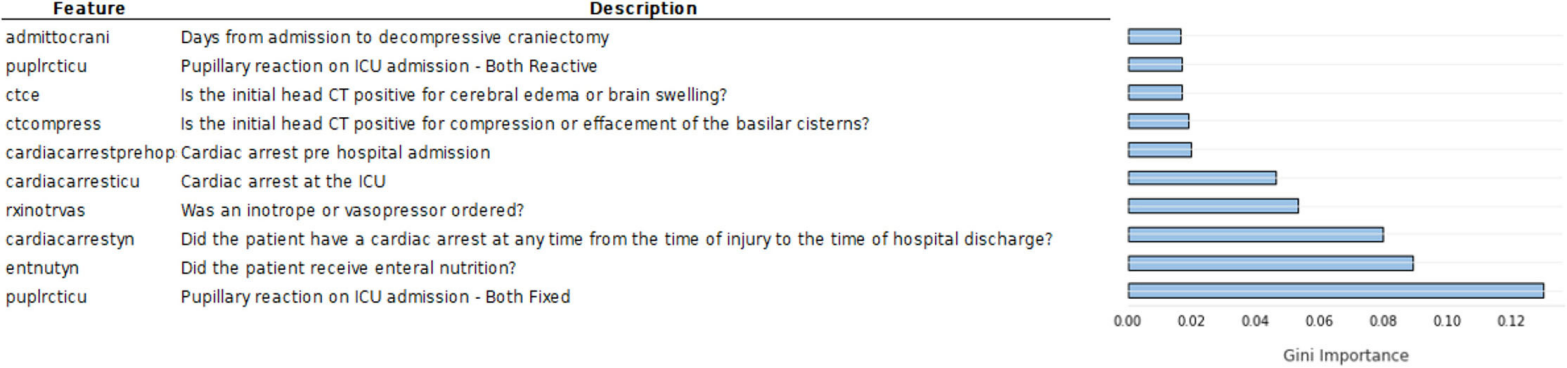
Ten most important features according to Gini feature importance computed by XGBoost.

From Figure 6, it is possible to see that it presents almost the same features as the RF computed feature importance, as expected. However, there are some differences, including the higher values for the first three features, indicating that XGBoost gave more importance to these features than RF.

Quantitatively, feature importance values are low in both methods, especially after the top five, indicating that the predictive value of those less important features does not vary much. Nevertheless, the top features present feature importance two times higher than the fifth to tenth top features, which is a considerable difference.

### 3.3. Clinical Considerations

As previously stated, it is of the utmost importance for physicians to understand algorithm results, therefore, we must analyze the results from the clinical point of view. Regarding the feature importance results obtained by Koerhsen's Feature Selector (Figure 4), enteral nutrition is the most relevant feature by a considerable margin. As enteral nutrition is used in critically ill patients, its correlation with mortality makes sense. However, we would expect other features such as midline shift, brain swelling, and subarachnoid hemorrhage to be of higher importance as they are directly related to the severity of the injury, i.e., the more severe the injury is, the higher damage to the skull and brain. Additionally, these CT related features are highly important in the current state-of-the-art TBI mortality prediction (15, 16). The high importance of other features like the number of days to the removal of the first and second catheter cannot be intuitively explained as there is no apparent direct connection between them and TBI. Placing catheters in patients is a common practice to allow easy access to the patient blood and facilitate the administration of pharmaceuticals. One possible explanation of this is if the catheters are removed early in the hospital stay, it can indicate that the patient does not require pharmaceuticals, and therefore, its condition is not severe, indicating a low chance of mortality. Nevertheless, these indirect connections are not easy to interpret and consequently may not be valuable to physicians. A similar interpretation can be made regarding the ordering of hypertonic saline. It is common practice to use hypertonic saline as a source of nutrients for hospitalized patients. If such practice is not required, it may indicate that the patient can eat and drink and therefore his condition may not be severe, reducing the chance of mortality.

The feature importance results obtained by the RF model (Figure 5) are overall more clinically relevant since no features related to catheter removal are in the top 10. Additionally, pupillary reactivity, namely when both pupils are fixed, is the most important feature, followed closely by enteral nutrition and the existence of cardiac arrest. Pupillary reactivity is very important in the currently commercialized models, CRASH (15) and IMPACT (16), as it is a direct indicator of the severity of the patient's brain injury, i.e., if both pupils are fixed, the damage is considerable and the patients (35, 36). Compression or effacement of basilar cisterns is also among the most important features, according to the RF model. This adds to the reliability of this method, as the compression of basilar cisterns has shown a high correlation with mortality (37). Nevertheless, there are still some features, apparently important for the RF prediction, that do not present a direct connection with the injury like the ordering of hypertonic saline and vasopressors.

**Figure 7:**
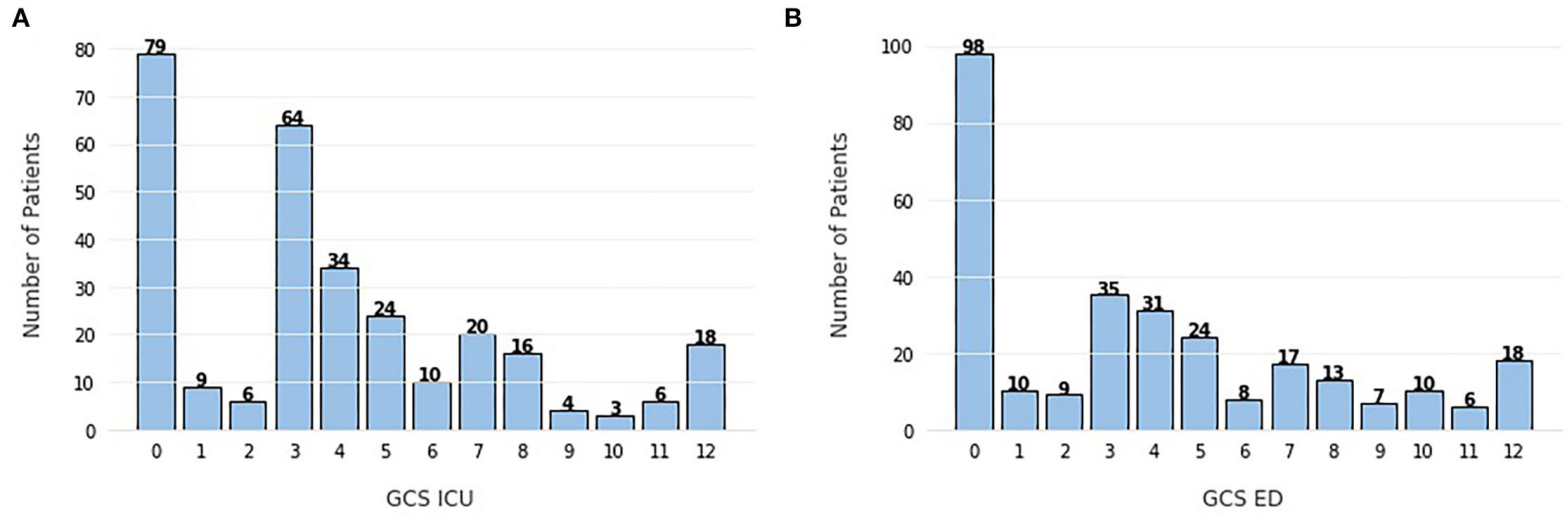
Frequency of Glasgow Coma Scale (GCS) scores **(A)** evaluated in the intensive care unit (ICU), **(B)** evaluated in the emergency department (ED). The scores range from 3 (completely unresponsive) to 15 (completely responsive).

In all three methods, there is an unforeseen absence of the GCS, which is a very valuable indicator of the patient's state and it is standard practice in brain injuries (36). In an attempt to explain this disregard for such a highly used feature in this task, we can look at Figure 7 and analyze the frequency of each score. It is clear that the majority of patients present a GCS score below 9, with the peak being at 3. The GCS ranges from 3, indicating a completely unresponsive patient, to 15, completely responsive. With this in mind, the cohort in this study is mostly constituted by very severe cases of TBI, therefore, it can be inferred that GCS scores should be a good indication of in-hospital mortality, which is not observed here.

The unexpected absence of CT related features such as midline shift and brain swelling in the top 10 features can be related to the previously referred heterogeneity of the condition in pediatric patients, due to their still developing brains. The pre-conceived importance of CT-based features is mostly based on adult cases, but eventually, in a cohort of pediatric patients where the brain and skull are still not fully formed, the importance of these CT and brain related features may be changed, which is why they are not visible in the feature importance figures. Nevertheless, this interpretation would still require confirmation by other external validation studies, with different datasets and methods of feature importance.

## 4. Limitations

This study presents some limitations that were identified and the possible strategies for future work. The size of the datasets in healthcare usually suffers from the lack of massive collections, which has been slowing the progress and application of AI solutions in the medical field. In the current study, the size of the dataset used is namely one of the limitations. The dataset may not cover the heterogeneity of the population, and eventually, the learning model did not generalize enough to cope with the variabilities of the population. On other hand, the small size of the dataset limited the approach to be used. Deep learning methods were not applied, since they need massive data to be trained. Moreover, the dataset only contains pediatric patients, which is a less studied group of the population for TBI-related classification. For some ages, there are only a few cases, which do not allow to study the correlation between some variables and age. Finally, the dataset did not contain continuous data, which could be very useful for mortality prediction and allow the creation of novel classification models that take into consideration the time sequence.

## 5. Conclusion

In this study, four machine learning methods were compared in terms of their ability to predict mortality after TBI. XGBoost seems to be the best performer of the tested models, achieving an AUC of 0.91, using no feature selection. The feature selector tool tested showed promising results for KNN and ANN, outperforming PCA and ICA. Decision tree-based methods performed better with no feature selection.

The comparison between different combinations of machine learning models and feature selection tools allows us to conclude that feature selection can improve prediction quality, either through external feature selection methods paired with models like KNN or with decision-tree based models with inherent feature selection capability, like XGBoost. Besides, feature selection also introduces more comprehensibility to the methods, facilitating the comparison of predictive value and clinical significance.

Regarding the feature importance, there are some differences between the expected clinically significant variables and important features for prediction, namely the absence in the top 10 of the GCS features and the CT-based features, which may be explained by the still developing brain and skull of pediatric patients, that causes a higher variety of outcomes in this cohort.

Exploring a new cohort of patients portrays challenges such as dealing with smaller datasets and less literature to compare results. This entails further responsibilities such as exploring different methods instead of improving or building upon a more established methodology. Nevertheless, despite the exploratory nature of this study, the results obtained showed that machine learning methods can take advantage of the information in ICU data, allowing the prediction of mortality in TBI pediatric patients with high accuracy.

Predictive tools can be helpful in the prognosis process by warning physicians about more critical cases and allowing them to adapt their medical care plan based on the severity of each case.

Future study may focus on training and testing these models on bigger and multicenter datasets, making them more robust, but also, focused on the initial period of the ICU admission which is the most critical time window for prognosis. For example, using only the data from the first two days of admission, in order to make a faster prediction that can more carefully guide medical attention. Efforts can also be made toward predicting not only mortality but also the functionality of the patient after a certain amount of time, providing physicians information that can lead to more focused and overall better medical care.

## Data Availability Statement

Publicly available datasets were analyzed in this study. This data can be found here: GitHub, https://github.com/fouticus/hptbi-hackathon.

## Author Contributions

JF conducted the experiments, AI-based model development, and testing. JF, XL, HO, and TP performed the analysis of the results. JF wrote the first draft of the manuscript. JF and XL performed the clinical interpretation of the results. All authors reviewed the manuscript. All authors contributed to the article and approved the submitted version.

## Conflict of Interest

The authors declare that the research was conducted in the absence of any commercial or financial relationships that could be construed as a potential conflict of interest.

## Publisher's Note

All claims expressed in this article are solely those of the authors and do not necessarily represent those of their affiliated organizations, or those of the publisher, the editors and the reviewers. Any product that may be evaluated in this article, or claim that may be made by its manufacturer, is not guaranteed or endorsed by the publisher.
